# 1556. Increase in New HIV Diagnoses Following Decrease in Use of Pre-Exposure Prophylaxis (PrEP) During the COVID-19 Pandemic

**DOI:** 10.1093/ofid/ofad500.1391

**Published:** 2023-11-27

**Authors:** Li Tao, J Carlo Hojilla, Valentina Shvachko, Juan Yang, Christoph C Carter, Moupali Das, Melanie de Boer

**Affiliations:** Gilead Sciences, Foster City, California; Gilead Sciences Inc, Foster City, California; Gilead Sciences, Inc., Foster City, California; Gilead Sciences, Foster City, California; Gilead Sciences, Foster City, California; Gilead Sciences, Foster City, California; Gilead Sciences, Foster City, California

## Abstract

**Background:**

Population-level longitudinal data evaluating trends in new HIV diagnoses and use of pre-exposure prophylaxis (PrEP) to assess the impact of COVID-19 on HIV prevention efforts are limited. Here, we evaluated changes in new HIV diagnoses in people who could benefit from PrEP (PWBP), PrEP use, and new HIV diagnoses among people prescribed PrEP prior to and during the COVID-19 pandemic in the United States.

**Methods:**

PWBP prescribed PrEP at least once after January 2012 were identified using a prescription claims database (IQVIA LRxDx). HIV-negative persons who had ever received emtricitabine(F)/tenofovir disoproxil fumarate or F/tenofovir alafenamide for PrEP between July 2019 and December 2022 were included in this analysis. PrEP use was defined as having an active prescription for oral PrEP before supply ended during the month. New HIV diagnoses per 100 persons per month (PPM) was calculated as new diagnoses divided by PWBP with an active enrollment status. Interrupted time series analysis with segmented regression was used to assess changes in the slope of PrEP use and HIV diagnosis before and during the pandemic.

**Results:**

Compared to January 2020 (pre-COVID), new HIV diagnoses in PWBP decreased by 44% in April 2020 and 34% in December 2020. New diagnoses subsequently increased each month, reaching pre-COVID levels in July 2021 (Figure 1). PrEP use similarly declined at the start of the pandemic, which was associated with a subsequent increase in new HIV diagnoses among persons in this group (Figure 2). However, PrEP use has increased steadily since November 2020, with a monthly increase of approximately 3600 persons (p< 0.01). As PrEP use increased, we observed a proportional decrease in new HIV diagnoses among people who were prescribed PrEP, with an average of 0.13 new diagnosis per 100 PPM, while new diagnosis in all PWBP increased with an average of 0.60 per 100 PPM during the same post-COVID period.

**Figure:**
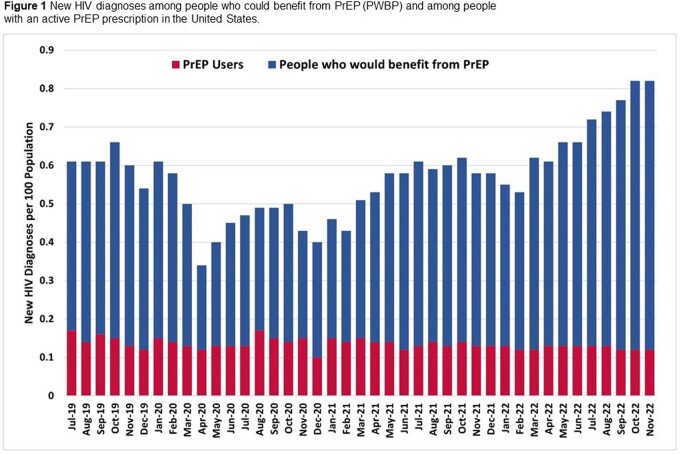

**Figure:**
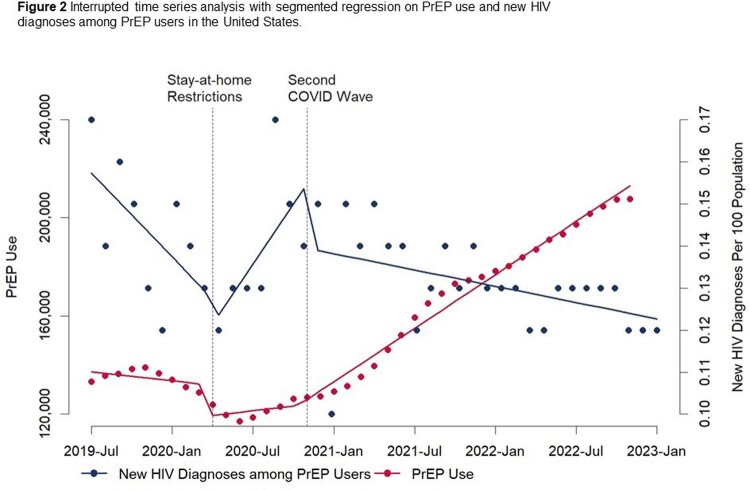

**Conclusion:**

Decreased HIV diagnoses in PWBP early in the pandemic coincided with stay-at-home restrictions and peaks in COVID-19 hospitalizations, which may have influenced sexual behaviors and HIV testing access. The decrease in PrEP use and subsequent increase in HIV diagnoses highlight the importance of sustained access to PrEP and other HIV prevention services during public health emergencies.

**Disclosures:**

**Li Tao, MD, PhD**, Gilead Sciences, Inc: Employee|Gilead Sciences, Inc: Stocks/Bonds **J Carlo Hojilla, RN, PhD**, Gilead Sciences, Inc: Employee|Gilead Sciences, Inc: Stocks/Bonds **Valentina Shvachko, MS**, Gilead Sciences, Inc: Employee|Gilead Sciences, Inc: Stocks/Bonds **Juan Yang, PhD**, Gilead Sciences, Inc: Employee|Gilead Sciences, Inc: Stocks/Bonds **Christoph C. Carter, MD, PhD**, Gilead Sciences, Inc: Employee|Gilead Sciences, Inc: Stocks/Bonds **Moupali Das, MD**, Gilead Sciences, Inc: Employee|Gilead Sciences, Inc: Stocks/Bonds **Melanie de Boer, PhD**, Gilead Sciences, Inc: Employee|Gilead Sciences, Inc: Stocks/Bonds

